# Hippocampal volume in affective and non-affective psychosis

**DOI:** 10.1016/j.schres.2025.06.021

**Published:** 2025-07-03

**Authors:** Katie Gibbs, Maureen McHugo, Alexandra Moussa-Tooks, Neil D. Woodward, Stephan Heckers, Maxwell J. Roeske

**Affiliations:** a Department of Psychiatry and Behavioral Sciences, Vanderbilt University Medical Center, Nashville, TN, USA; b Department of Psychiatry, University of Colorado Anschutz Medical Campus, Aurora, CO, USA; c Department of Psychological and Brain Sciences, Indiana University Bloomington, Bloomington, IN, USA; d Program in Neuroscience, Indiana University Bloomington, Bloomington, IN, USA

**Keywords:** Hippocampus, Psychosis spectrum, Neuroimaging, Incomplete hippocampal inversion

## Abstract

Hippocampal volume change in non-affective psychotic disorders begins in the anterior region and spreads to the posterior region as the illness progresses. It is unclear whether similar hippocampal volume changes are present in affective psychosis. Here, we test the hypothesis that anterior and posterior hippocampal volumes are differentially affected in affective psychosis. We also investigated the prevalence of a marker of atypical hippocampal development, incomplete hippocampal inversion (IHI), and its impact on hippocampal volume in affective psychosis. We analyzed total, anterior, and posterior hippocampal volumes using automated segmentation of structural MRI data from 103 affective psychosis, 242 non-affective psychosis, and 214 healthy control individuals. Compared to healthy participants, individuals with affective psychosis exhibited smaller posterior hippocampal volumes. The two psychosis patient groups did not differ significantly in total or regional hippocampal volumes. IHI prevalence and severity were greater in individuals with non-affective and affective psychosis. Our findings suggest hippocampal volume is reduced in the posterior, but not anterior, hippocampus in affective psychosis and that IHI may be a marker for an increased risk of developing psychosis.

## 1. Introduction

Hippocampal volume reduction in schizophrenia is one of the most consistent and robust anatomical brain abnormalities in psychiatric illnesses (Okada et al., 2023; Van Erp et al., 2016). Post-mortem (Roeske et al., 2021a) and neuroimaging (Adriano et al., 2012; Haijma et al., 2013) meta-analyses confirm hippocampal volume abnormalities in patients at various illness stages. Hippocampal volume is already smaller in individuals experiencing their first episode of psychosis (Adriano et al., 2012) and decreases with illness chronicity (Velakoulis et al., 2006). Recent evidence demonstrates that the initial reductions in volume are localized to the anterior region of the hippocampal longitudinal axis (i.e., the hippocampal head) (Chopra et al., 2023; Kalmady et al., 2017; McHugo et al., 2020, 2024) and spread to the posterior region (i. e., the hippocampal body and tail) as the illness progresses (McHugo et al., 2018). These results suggest hippocampal volume reductions in schizophrenia have a specific temporal and spatial pattern.

It is unclear if individuals experiencing psychosis but not meeting the criteria for a primary psychotic disorder also demonstrate this pattern of hippocampal volume reductions. The transdiagnostic psychosis spectrum model suggests that affective psychoses, defined as psychosis within a major mood episode (i.e., major depressive disorder (MDD) or bipolar disorder), and non-affective psychoses (i.e., schizophrenia, schizophreniform, schizoaffective disorder) are distinct phenotypes that exist on a continuum of illness (Cattarinussi et al., 2023; Guloksuz and Van Os, 2018). The continuum model is supported by numerous studies demonstrating similarities between genetics (Amare et al., 2019; Lee et al., 2013; Rasic et al., 2014; Schulze et al., 2014), cognition (Bora et al., 2009; Kristian Hill et al., 2013), and neuroanatomy (Goodkind et al., 2015; Ivleva et al., 2013) across the psychosis spectrum. Evidence suggests hippocampal morphology can serve as a marker to differentiate between diagnostic classes (del Re et al., 2021). Identifying patterns of hippocampal volume alterations across the psychosis continuum may enable opportunities for prognostication and early intervention to prevent illness progression and functionally meaningful volume loss.

Studies consistently suggest that hippocampal volume reduction is a shared feature across the psychosis continuum (Birur et al., 2017; Brown et al., 2011; Cao et al., 2023; Hartberg et al., 2011; Mathew et al., 2014). Most studies report that volume loss is greater in schizophrenia compared to bipolar disorder (Brown et al., 2011; Knöchel et al., 2014; Sato et al., 2021) and depression (Ota et al., 2017; Shi et al., 2022), including studies of individual hippocampal subfields (Cao et al., 2023; Haukvik et al., 2015; Mathew et al., 2014). However, these studies are difficult to interpret because few report on the presence or severity of psychosis in the bipolar disorder or depression groups, and some limit patient comparisons to healthy controls and not to other patient groups. Only one study has directly investigated hippocampal volume along the longitudinal axis in individuals with affective psychosis. Del Re et al. found smaller anterior hippocampal volume in groups of individuals with schizophrenia and psychotic bipolar disorder when compared to healthy controls (del Re et al., 2021). This study also reported differences in posterior hippocampal volume in individuals with schizoaffective disorder or schizophrenia compared to controls but not in individuals with psychotic bipolar disorder. These findings have not been replicated in other studies to date.

Abnormalities in hippocampal development contribute to hippocampal volume deficits. The transdiagnostic continuum model of psychosis suggests that affective and non-affective psychoses are different phenotypic expressions of a shared pathogenesis, and differentiation may occur with specific disease modifiers (Guloksuz and Van Os, 2018). For example, Murray et al. highlight that while schizophrenia and bipolar disorder have shared symptoms and susceptibility genes, only schizophrenia is linked to perinatal complications that impair neurodevelopment, particularly medial temporal lobe structures (Murray et al., 2004). Incomplete hippocampal inversion (IHI) is a developmental medial temporal lobe abnormality that occurs during the second trimester of perinatal development (Bajic et al., 2010). IHI is more prevalent and severe in individuals with non-affective psychosis than healthy individuals (Roeske et al., 2021b), and it is correlated with both hippocampal volume reductions and shape deformations (Roeske et al., 2022). The prevalence of IHI and whether IHI differentially impacts the hippocampus in affective psychoses is unknown. Studying IHI allows us to test the hypothesis that affective and non-affective psychosis share a common pathogenesis that may be differentiated by abnormal development.

Here, we test the hypothesis that the differences in hippocampal volume are less pronounced in affective psychosis compared to non-affective psychosis. We first examine whether total hippocampal volume differs by group. Based on prior findings, we expect that individuals with affective psychosis will display larger total hippocampal volumes compared to non-affective psychosis, but smaller volumes compared to healthy controls. Next, we test whether there are region-specific volume differences by measuring volume in the anterior and posterior regions of the hippocampus across groups. Based on findings recently reported by del Re et al., we anticipate volume deficits in the anterior hippocampus in the affective group compared to healthy controls. After, we investigate if IHI explains differences in volume by assessing IHI prevalence and severity within affective psychosis. Finally, we test whether our findings are consistent with the established gradient of hippocampal volume reductions across the psychosis spectrum using illness chronicity (i.e., healthy control > first episode affective psychosis > chronic affective psychosis > first episode non-affective psychosis > chronic non-affective psychosis).

## 2. Methods

The Vanderbilt University Institutional Review Board approved this study. All participants provided written informed consent.

### 2.1. Participants

We analyzed data from 559 individuals (103 persons with an affective psychotic disorder, 242 persons with a non-affective psychotic disorder, and 214 persons without a major psychiatric disorder) matched on age and parental education (Table 1). Patients with schizoaffective disorder were included in the non-affective psychosis group, consistent with previous literature investigating hippocampal volume deficits across the psychosis spectrum (del Re et al., 2021; Velakoulis et al., 2006). Forty-one of the 46 patients with schizoaffective disorder also met DSM-IV criteria for schizophrenia, based on evaluation of their clinical data. The remaining five cases lacked necessary information on the degree of functional impairment (DSM-IV criterion B) or length of illness (DSM-IV criterion C) to confidently confirm that they also met all criteria for schizophrenia. Patients were recruited from psychiatric inpatient and outpatient clinics of the Vanderbilt University Medical Center Psychotic Disorders Program as part of one of three studies (CT00762866, R01MH070560, or R01MH102266).

Of the 559 persons reported here, 366 persons (199 with a non-affective psychotic disorder, 167 without a major psychiatric disorder) had been included in previous reports of hippocampal volume and IHI (McHugo et al., 2018, 2020, 2024; Roeske et al., 2021b, 2022). 103 persons with an affective psychotic disorder had previously been included in analyses of other brain regions (Moussa-Tooks et al., 2022), but hippocampal volumes are presented here for the first time.

Healthy control participants were recruited from the community through advertisements. Diagnostic assessment was completed with the Structured Clinical Interview for DSM-IV (First et al., 2002). Additional clinical symptoms were measured with the Positive and Negative Syndrome Scale (PANSS; Kay et al., 1987), the Hamilton Depression Rating Scale (HAMD; Hamilton, 1960), and the Young Mania Rating Scale (YMRS; Young et al., 1978). Premorbid IQ was measured with the Wechsler Test of Adult Reading (WTAR; Wechsler, 2001). Participants were excluded for significant medical illness, head injury, pregnancy, meeting criteria for substance abuse within the past month for schizophrenia participants (three months in the case of R01MH102266) or lifetime history of substance abuse/dependence in controls, or age under 16 or over 65.

Healthy controls were excluded if they had a current or past psychiatric illness, current psychotropic drug use, or a first-degree relative with a psychotic disorder. All participants had a T1-weighted MRI scan without motion artifacts. To test the hypothesis that duration of illness impacts hippocampal volume, the patient cohort was divided into chronic (duration of illness ≥2 years) and early (duration of illness <2 years) subgroups within both non-affective (chronic *N* = 118, early *N* = 124) and affective (chronic *N* = 52, early *N* = 51) groups.

### 2.2. Structural MRI data acquisition and processing

Structural imaging data was acquired on a 3 T Philips Intera Achieva scanner at the Vanderbilt University Institute of Imaging Sciences (Philips Healthcare, Inc.). 39 persons with affective psychosis, 109 with non-affective psychosis, and 104 healthy control participants recruited in study R01MH070560 received a 3D T1-weighted scan (voxel resolution: 1 mm^3^; field of view = 256^2^; number of slices = 170; TE = 3.7 ms; TR = 8.0 ms). The remaining participants recruited in study R01MH102266 underwent a T1-weight scan with slightly different scan parameters (voxel resolution: 1 mm^3^; field of view = 256^2^; number of slices = 170; TE = 4.6 ms; TR = 8.9 ms). Participants were included after each image was visually inspected and determined to be without motion or other artifacts. Participants who had not been included in previous hippocampal analyses (*N* = 193 total; 103 affective psychosis, 43 non-affective psychosis, 47 controls) required visual inspection prior to analyses. This resulted in removing 4 participants (Schizophrenia = 1, Schizophreniform = 1, Bipolar = 2) from regional, but not whole, hippocampal volume analyses. For IHI scoring, all images were reoriented toward the MNI152 atlas using FSL rigid body transformation (Jenkinson et al., 2002).

Each image was processed using the Freesurfer 6 (Dale et al., 1999; Fischl et al., 2002) hippocampal subfield segmentation module (Iglesias et al., 2015). For volume comparisons along the hippocampal longitudinal axis, anterior (head) and posterior (body + tail) volume composites were created by combining Freesurfer 6 hippocampal subfield segmentations. We defined the anterior hippocampus as the sum of the following subfield volumes within the hippocampal head: CA1, CA3, CA4, molecular layer, GC/DG, subiculum, and presubiculum. We defined the posterior hippocampus as those same subfields within the hippocampus body and tail.

Each participant’s subfield segmentation was visually inspected for errors, including tissue outside the hippocampus or incomplete labeling of the hippocampus. We identified failed automated segmentations in 63 participants. All were manually corrected using ITK-SNAP version 3.8.0 (Yushkevich et al., 2006).

### 2.3. IHI scoring

The criteria for assessing IHI in this study were previously published and validated (Cury et al., 2015). IHI scores for each hippocampus ranged from 0 to 8.5. IHI was present for scores of ≥4. IHI was assessed by two observers, with a portion of the sample (*N* = 366) previously scored and published with very strong (kappa = 0.88) intraobserver and substantial (kappa = 0.76) interobserver reliability (Roeske et al., 2021b). The remaining participants received IHI scores following training. All criteria were assessed in the coronal view for both the left and right hippocampus using ITK-SNAP (Version 3.8.0).

### 2.4. Statistical analysis

We analyzed data using linear mixed models with the R packages lme4 (Bates et al., 2015), emmeans (Lenth, 2018), and car (Fox et al., 2014). First, to test for total hippocampal volume deficits, we fit a linear mixed model predicting volume from the fixed effects of group (non-affective, affective, control), hemisphere (left, right), and their interactions, adjusting for estimated total intracranial volume (ICV), age, and sex with participant as a random effect. To test for differences in volume along the longitudinal axis of the hippocampus, we used the same model but also included the fixed effect of region (anterior, posterior) and its interactions. We conducted an analysis of variance (ANOVA) significance test on the fixed effects for all model outputs. For all statistical tests, the significance level was defined as alpha <0.05. Significant effects were followed by pairwise contrasts adjusted for multiple comparisons with Bonferroni correction.

To test IHI prevalence across groups, we performed a χ^2^ test on the IHI threshold score (≥4) for each hemisphere. To test group differences in IHI severity (greater total IHI score being more severe), we performed a *t*-test on the total IHI score (0–10) for each hemisphere. We also performed follow-up χ^2^ and *t*-tests comparing prevalence and severity between healthy individuals and all individuals experiencing psychosis (i.e., a single group of affective and non-affective psychosis). To examine the effect of IHI on total hippocampal volume, we conducted a two-step sensitivity analysis using linear mixed models. In step 1, we used the models previously mentioned (Volume Model, Step 1: Volume ~ Group x Hemisphere + ICV + Age + Sex + (1∣Participant)). In step 2, we added IHI as a fixed effect to the model to test if IHI contributes to volume (Volume Model, Step 2: Volume ~ Group x Hemisphere + IHI + ICV + Age + Sex + (1∣Participant)). Marginal *R*^2^ and AIC were used to assess model fit for models in Step 1 and Step 2. This approach was first conducted for whole hippocampal volume and then repeated for regional hippocampal volume analysis.

### 2.5. Exploratory analyses

We investigated the impact of duration of illness, chlorpromazine equivalents, and lifetime incidence of cannabis and alcohol use on hippocampal volume by including each variable as a covariates into the models outlined above. These variables are listed in Table 1.

## 3. Results

### 3.1. Whole hippocampal volume differences

**Fig. 1.**
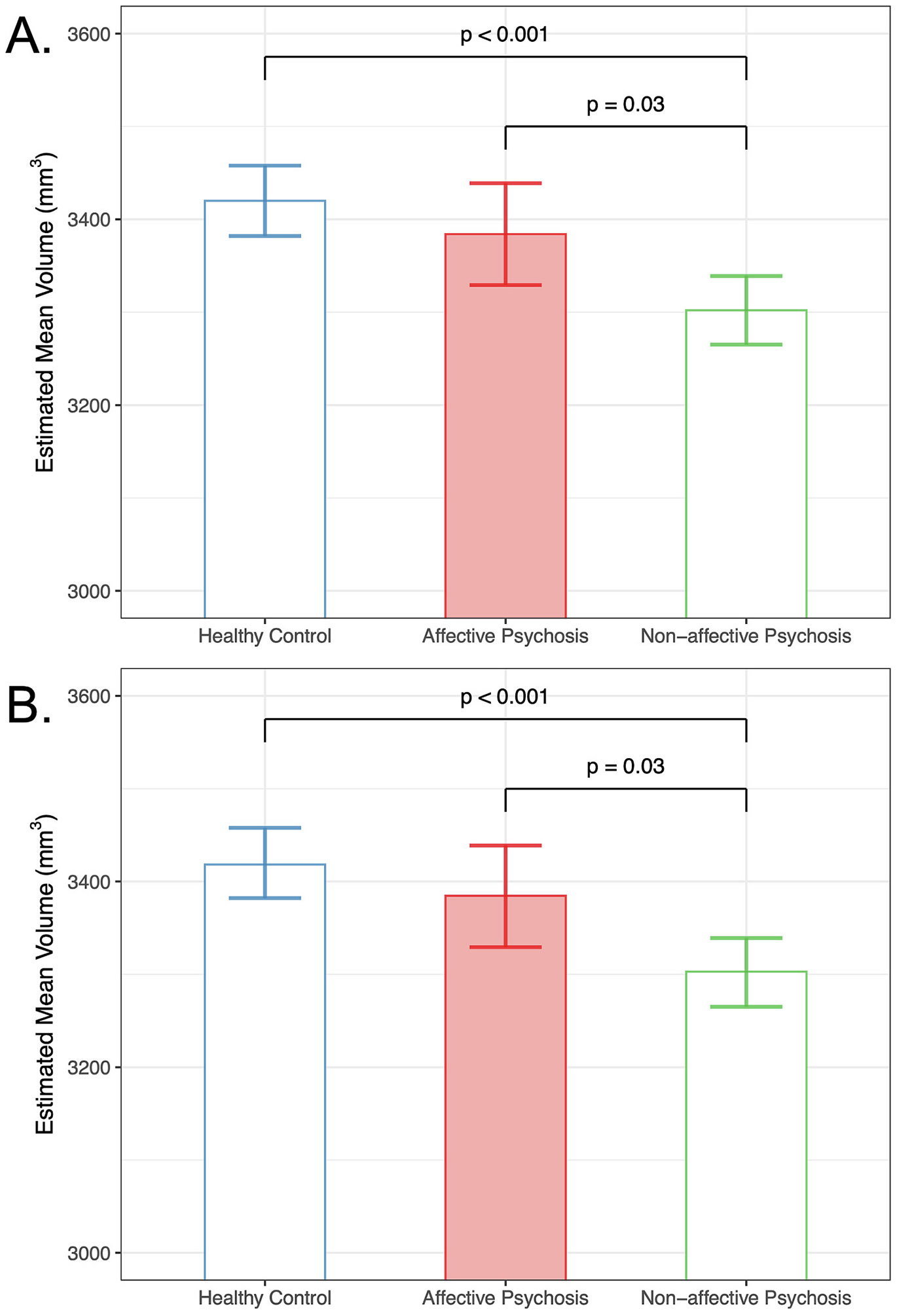
Whole hippocampal volume by group. A. In persons with non-affective psychosis, whole hippocampal volume is smaller relative to both controls and affective psychosis. B. When IHI is included in the model as a fixed effect, whole hippocampal volume is still smaller in non-affective psychosis compared to both controls and affective psychosis. Significant effects followed Bonferroni correction for multiple comparisons. Error bars indicate 95 % confidence intervals of the estimated marginal mean volumes.

We tested for group differences in total hippocampal volume. Without IHI in the model, we observed a significant main effect of group (F(2,551) = 11.78, *p* ≤0.001) and no significant interactions of group by hemisphere. The affective psychosis group did not differ from the controls group (*p* > 0.05; Fig. 1A). The non-affective group displayed smaller total hippocampal volume compared to the affective psychosis (t(551) = 2.59, *p* = 0.03) and control (t(551) = 4.77, *p* < 0.0001) groups.

### 3.2. Incomplete hippocampal inversion

In our sample of 555 individuals (100 affective, 241 non-affective, 214 control), we found 98 (18 % of total sample) unilateral left IHI (23 affective, 45 non-affective, 30 control), 30 (5 %) bilateral IHI (4 affective, 18 non-affective, 8 control), and 8 (1 %) unilateral right IHI (1 affective, 4 non-affective, 3 control) cases. The remaining 419 participants (76 %) showed no IHI. Four participants were excluded due to failed processing of the images (3 affective, 1 non-affective).

Across all groups, IHI in the left hemisphere did not differ in prevalence (27 % in affective, 26 % in non-affective, 18 % in healthy control groups; χ^2^ = 5.56, *p* = 0.06; Table 2) or severity (total IHI score of 2.95 in affective, 2.90 in non-affective, 2.64 in healthy control groups; *t* = 2.59, *p* = 0.07). Prevalence and severity did not differ across all diagnostic groups in the right hippocampus. Combining individuals with affective and non-affective psychosis into a single psychosis group (*n* = 341) and comparing them to the control group revealed a significant increase in left IHI prevalence (χ^2^ = 5.05, *p* = 0.02) and severity (*t* = 2.35, *p* = 0.02), but no differences in right IHI prevalence or severity.

Including IHI as a fixed effect in the linear model for total hippocampal volume, we again observed a significant main effect of group (F(2, 551.35) = 11.33, *p* < 0.001) and no significant interactions of group by hemisphere. We also observed a main effect of IHI (F(1, 938.49) = 5.17, p = 0.02). Pair-wise comparisons confirmed that with IHI in the model, no differences exist between affective psychosis and control groups (*p* > 0.05; Fig. 1B), and the non-affective psychosis group continued to show smaller total hippocampal volume compared to affective psychosis (t(551) = 2.58, *p* = 0.03 and control (t(552) = 4.67, p < 0.0001) groups. Including IHI improved the Volume Model fit for whole hippocampal volume marginally (Step 1: R^2^ = 0.520, AIC = 15,333.6; Step 2: R^2^ = 0.521, AIC = 15,325.06; Table 3).

### 3.3. Regional hippocampal volume differences

**Fig. 2.**
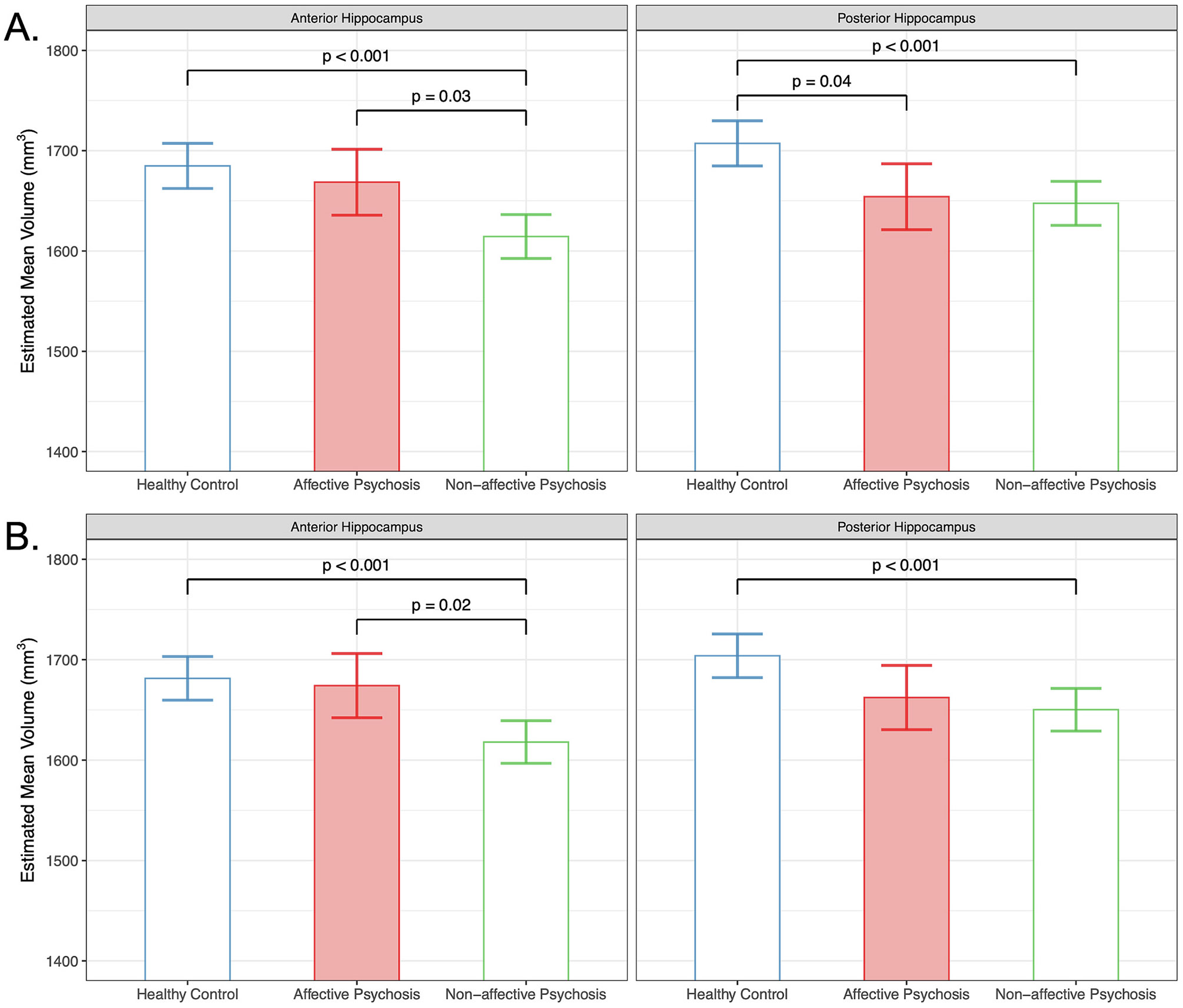
Regional hippocampal volume by group. A. In persons with non-affective psychosis, hippocampal volume is smaller in both anterior and posterior regions relative to controls. In persons with affective psychosis, only posterior hippocampal volume is smaller relative to controls. The affective group also displayed larger anterior hippocampal regions relative to the non-affective group. B. When IHI is included in the model as a fixed effect, hippocampal volume is still smaller in both anterior and posterior regions in the non-affective group relative to controls. The affective group still exhibits larger anterior hippocampal regions relative to the non-affective group. Persons with affective psychosis do not differ from controls. Significant effects followed Bonferroni correction for multiple comparisons. Error bars indicate 95 % confidence intervals of the estimated marginal mean volumes.

We next examined regional hippocampal volume differences by group. Without IHI in the model, we found a significant group by region interaction (F, 1656) = 5.56, *p* < 0.01) in the context of a main effect of group (F(2, 547) = 11.27, *p* < 0.001). The affective psychosis group differed from the control group only in the posterior hippocampus (t (736) = 2.75, *p* = 0.04). The affective psychosis group had larger anterior (t(735) = 2.84, p = 0.03) hippocampal volumes than non-affective psychosis. The non-affective psychosis group had smaller anterior (t(743) = 4.74, p < 0.001) and posterior (t(743) = 4.03, p < 0.001) hippocampal volumes compared to controls (Fig. 2A). We did not observe a significant group by region by hemisphere interaction.

Including IHI in the model again revealed a significant main effect of group (F(2, 544.60) = 9.90, p < 0.001) and group by region interaction (F(2, 1646.09) = 4.84, p < 0.01), as well as a significant main effect of IHI (F(1, 2160.19) = 98.81, p < 0.001). The non-affective psychosis group still showed smaller posterior volumes compared to controls (t(750) = 3.74, p < 0.001), but including IHI in the model removed the significant posterior volume difference between affective psychosis and control groups (t(743) = 0.39, *p* > 0.05; Fig. 2B). We again observed smaller anterior hippocampal volume in the non-affective psychosis group compared to both control (t(750) = 4.42, p < 0.001) and affective psychosis (t(740) = 3.03, *p* = 0.02) groups. Including IHI in the model did not reveal a significant group by region by hemisphere interaction (p > 0.05), but did improve the Volume Model fit for regional hippocampal volume (Step 1: R^2^ = 0.39, AIC = 28,479.11; Step 2: R^2^ = 0.42, AIC = 28,231.3; Table 3).

### 3.4. Volume differences by illness stage and diagnosis

**Fig. 3.**
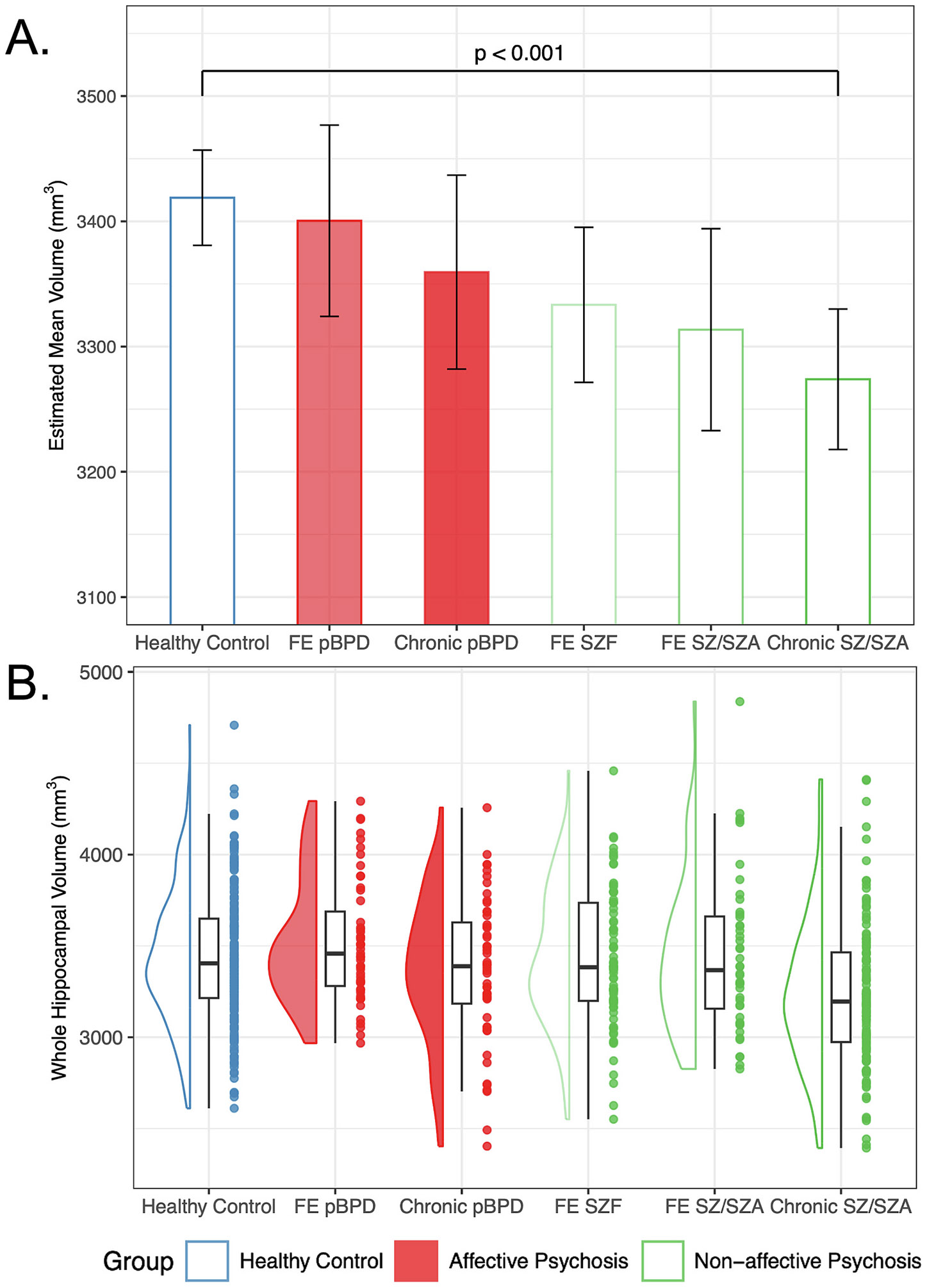
Whole hippocampal volume by illness stage and disorder. A. Estimated mean hippocampal volumes based on model output. The magnitude of hippocampal volume differences increases across the psychosis spectrum (non-affective psychosis > affective psychosis) and by stage of illness (chronic > first episode). Only the chronic schizophrenia/schizoaffective disorder group displayed significantly smaller hippocampal volumes relative to controls. Error bars indicate 95 % confidence intervals of the estimated marginal mean volumes. B. Modified violin plot, box plot, and individual whole hippocampal volumes included in the linear mixed model to generate estimated mean volumes. FE = First Episode; pBPD = Psychotic Bipolar Disorder; SZF = Schizophreniform Disorder; SZ = Schizophrenia; SZA = Schizoaffective Disorder; increasing color saturation indicates greater illness duration across groups.

Examining volume differences across illness stages and diagnoses, we observed a significant main effect of diagnosis (F(5, 548) = 5.14, p < 0.001) and no significant diagnosis by hemisphere interactions (F(5, 553) = 1.22, p > 0.05). Follow-up tests revealed that only the chronic schizophrenia/schizoaffective cohort showed reduced whole hippocampal volume compared to the control group (t(548) = − 4.52, *p* = 0.0001; all other p’s > 0.05; Fig. 3).

### 3.5. Exploratory analyses

Including duration of illness, chlorpromazine equivalents, and lifetime incidence of alcohol or cannabis use disorders into our models did not change the results or reveal new significant effects.

## 4. Discussion

We investigated hippocampal volumes in persons with affective and non-affective psychosis and found evidence for a gradient of volume reductions across the psychosis spectrum. Patients with affective psychosis demonstrate volume reductions in posterior, but not anterior, hippocampal regions, only if one accounts for IHI, a variant of hippocampal development. As reported earlier and consistent with prior literature, our sample of persons with non-affective psychosis showed reductions in anterior, posterior, and total hippocampal volumes (McHugo et al., 2018, 2020). Finally, there is a gradient of hippocampal volume loss across psychosis clinical phenotypes, but only persons with chronic schizophrenia or schizoaffective disorder have significantly reduced hippocampal volumes compared to healthy controls.

The current study furthers our understanding of hippocampal structure in psychosis by reporting a unique pattern of hippocampal volume deficits in affective psychosis. Whole and anterior hippocampal volumes in affective psychosis do not differ from healthy individuals and are significantly larger than patients with non-affective psychosis. Posterior hippocampal volumes in affective psychosis are smaller when compared to healthy individuals but do not differ from patients with non-affective psychosis. Recent models of hippocampal circuitry associate the anterior hippocampus with affective function and the posterior hippocampus with cognitive function (Genon et al., 2021; Poppenk et al., 2013; Strange et al., 2014). In this context, it is unexpected that we found hippocampal volume changes in affective psychosis only in the posterior hippocampus. However, this mapping of hippocampal functions conflicts with the previous finding of anterior hippocampal volume changes in the prodrome and early stages of non-affective psychosis when cognitive changes are already prominent. Ultimately, a better understanding of the hippocampal anterior/posterior gradient will help clarify hippocampal structure and function deficits in psychosis.

The pattern of hippocampal volume deficits reported here is inconsistent with the volumetric findings reported in the only other study of the hippocampal longitudinal axis across the psychosis spectrum (del Re et al., 2021). Our results suggest that anterior volumetric deficits, repeatedly observed in schizophrenia, are specific to non-affective psychosis. In contrast, del Re et al. report this pattern is also present in affective psychosis. Further, both studies demonstrate posterior volume differences between affective and non-affective psychosis. However, our study suggests posterior volumes are decreased in affective psychosis, and del Re et al. suggest they are decreased in non-affective psychosis. Differences in the study samples and data analysis may contribute to differing results. Our study sample is younger and includes individuals in the first two years of illness (50 % of individuals in affective psychosis group; 51 % of individuals in non-affective psychosis group). A sample that is older or has a greater duration of illness is likely to be associated with greater hippocampal volume loss and extension into the posterior hippocampus. Further, in the del Re study, individuals with poor-quality automated segmentation were removed from the analysis. In our study, we manually corrected poor segmentation to maximize individuals in our analysis. Our previous work has demonstrated that Freesurfer segmentation has a higher failure rate in severe IHI cases, which contributes to reductions in hippocampal volume (Roeske et al., 2021b). Therefore, eliminating failed hippocampal segmentations may artificially inflate reported volumes for groups with a greater IHI prevalence.

The prevalence and severity of left IHI are significantly greater in all patients with psychosis when compared to healthy controls. We did not detect a difference in IHI prevalence or severity across all three groups but observed a pattern suggesting potential differences in the left hemisphere that warrants further investigation. The relatively smaller sample size in the affective psychosis group and similarity in IHI features across both psychosis groups likely account for the inability to detect a significant main effect across all three groups. These results suggest that the presence of IHI may not be specific to one diagnosis but rather a marker of atypical brain development consistent with a developmental subgroup at an increased risk for developing psychosis. This is consistent with prior literature documenting that IHI is more prevalent in epilepsy (Mutti et al., 2021), in which individuals have a significant increase in risk of experiencing psychosis (Clarke et al., 2012; Thapa et al., 2024), but not in affective disorders without psychosis (Colle et al., 2016). Accounting for IHI did not change the results of the volumetric analysis except in the case of the reduced posterior hippocampal volumes for individuals with affective psychosis. Future studies should continue to characterize the IHI prevalence in populations without psychosis, including primary affective disorders without psychotic features, to clarify whether IHI is a reliable marker for psychosis.

We demonstrate that the magnitude of hippocampal volume loss increases across the psychosis spectrum (greater in non-affective psychosis) and by stage of illness (greater in chronic psychosis). This result replicates seminal studies (Velakoulis et al., 1999, 2006) and builds upon a robust literature relating hippocampal volume deficits to psychosis. Current models posit that hippocampal volume loss is the consequence of an excitation-inhibition imbalance within hippocampal microcircuitry that leads to hippocampal hyperactivity and a spreading excitotoxic effect (Briend et al., 2020; Kraguljac et al., 2013; Schobel et al., 2013). Previous work indicates that hyperactivity is a marker of acute illness state rather than an enduring feature of the illness (McHugo et al., 2022), suggesting that individuals with greater symptom burden of psychosis will have greater hippocampal volume loss. Longitudinal studies (Lieberman et al., 2018) and recent mega-analytic (Jiang et al., 2023, 2024) and large case-control (Chopra et al., 2023) studies of patients with psychosis demonstrate that the hippocampus plays a critical role in the origins of gray matter volume reductions that spread from the hippocampus to other regions of the hippocampal circuit, supporting this excitotoxicity mechanism. Therefore, reducing hippocampal hyperactivity to prevent or slow the loss of gray matter volume is a promising treatment target in psychosis (Tregellas, 2014; Uliana et al., 2024). Recent studies suggest that hippocampal hyperactivity can be pharmacologically targeted (Goff et al., 2023; Roeske et al., 2023). The magnitude of hippocampal volume loss across the psychosis spectrum and chronicity of illness reported here support this model: individuals with a greater symptom burden of psychosis (i.e., patients with non-affective psychosis) and individuals with greater chronicity of illness have smaller hippocampal volumes.

The strengths of our study include using a dimensional, large sample of individuals spanning the psychosis spectrum. Further, we examined the hippocampus across the longitudinal axis using automated hippocampal segmentation, aided by manual corrections to provide unbiased hippocampal volume estimates. However, this study has several limitations. First, the affective psychosis group does not include individuals with MDD with psychotic features. Second, we did not assess hippocampal subfields in this study. Third, groups were not fully matched on all demographic features including sex. Fourth, we could not assess the cumulative exposure of substances, including cannabis and alcohol, on hippocampal volume. Although participants with concurrent diagnoses of alcohol or cannabis use disorder were excluded from this study, we collected the lifetime history of substance use disorders in this cohort (Table 1). Covarying for lifetime alcohol and cannabis use disorder or dependence in our analyses did not change he results of our analyses. Lastly, the sample is cross-sectional. Future longitudinal studies are needed to investigate the progression of volume deficits.

In summary, our study finds evidence for a difference in affective and non-affective psychosis in both anterior and total hippocampal volumes. Our study also supports novel evidence for a posterior hippocampal difference in non-affective psychosis compared to healthy controls. These results support using a dimensional approach to identify disease characteristics for prognostication and development of novel treatment targets.

## Figures and Tables

**Table 1:** Participant demographics and clinical characteristics.

	Healthy Control		Affective Psychosis		Non-affective Psychosis			
	*N* = 214		*N* = 103		*N* = 242			
	Mean	SD	Mean	SD	Mean	SD	Statistic (t)	p
Age (years)	28.8	10.1	30.62	11.8	29.1	11.2	0.999	0.37
Parental education (years)	14.5	2.36	14.8	2.25	14.4	2.75	0.956	0.38
WTAR	111.1	10.83	106.6	12.6	99.8	15.79	39.42	<0.001
Duration of illness (years)	–	–	6.6	8.9	8.0	11.0	1.3	0.20
CPZE	–	–	284.1	158.7	437.9	590.2	3.4	<0.001
PANSS								
Positive	–	–	14.95	8.84	17.95	7.91	7.784	<0.001
Negative	–	–	10.34	3.61	15.6	6.92	28.41	<0.001
General	–	–	26.55	7.07	31.5	8.46	18.22	<0.001
	N		N		N		Statistic (X^2^)	p
Sex (Male/Female)	131/83		52/51		169/73		12.058	<0.01
Race (White/Black/Other)	150/51/13		83/11/9		152/80/10		24.889	<0.001
AUD	17		40		71		47.6	<0.001
CUD	7		38		107		102.2	<0.001
Illness Stage (Chronic/Early)	–		52/51		118/124			
Diagnosis								
Schizophrenia	–		–		116			
Schizoaffective disorder	–		–		46			
Schizophreniform	–		–		80			
Psychotic bipolar disorder	–		103		–			

**Table 2:** IHI prevalence and severity.

	Healthy		Affective		Non-affective			
	Control		Psychosis		Psychosis			
	N = 214		*N* = 100		*N* = 241			
	N	%	N	%	N	%	Statistic (X^2^)	p
Left IHI	38	18	27	27	63	26	5.56	0.06
Right IHI	11	5	5	5	22	9	3.48	0.17
	Mean	SD	Mean	SD	Mean	SD	Statistic (t)	p
Left IHI Score	2.64	1.2	2.95	1.32	2.9	1.55	2.59	0.07
Right IHI Score	2.04	0.9	2.14	1.04	2.16	1.07	1.48	0.23

**Table 3:** Sensitivity analysis of whole and regional hippocampal volume.

Whole Hippocampal Volume (mm^3^)					
Predictor	*F*	df	*p* value	*R*2	AIC
*Step 1: model without IHI*				0.52	15,333.6
Group	11.78	2, 551	< **0.001**		
Hemisphere	302.31	1, 556	< **0.001**		
ICV	255.49	1, 551	< **0.001**		
Age	1.16	1, 551	0.28		
Sex	0.07	1, 551	0.78		
Group:Hemisphere	0.95	2, 556	0.39		
*Step 2: model with IHI*				0.521	15,325.06
Group	11.33	2, 551.35	< **0.001**		
Hemisphere	223.06	1, 613.25	< **0.001**		
IHI	5.17	1, 938.49	< **0.05**		
ICV	257.92	1, 551.87	< **0.001**		
Age	1.12	1, 550.57	0.29		
Sex	0.06	1, 550.60	0.8		
Group:Hemisphere	1.11	2, 555.48	0.33		
Regional Hippocampal Volume (mm^3^)					
Predictor	*F*	df	p value	*R*2	AIC
*Step 1: model without IHI*				0.39	28,479.11
Group	11.27	2, 547	< **0.001**		
Hemisphere	264.03	1, 1656	< **0.001**		
Region	15.7	1, 1656	< **0.001**		
ICV	206.63	1, 547	< **0.001**		
Age	0.13	1, 547	0.72		
Sex	1.18	1, 547	0.28		
Group:Region	5.56	2, 1656	< **0.01**		
Group:Hemisphere: Region	1.5	2, 1656	0.22		
*Step 2: model with IHI*				0.42	28,231.3
Group	9.9	2, 544.6	< **0.001**		
Hemisphere	120.95	1, 1811.52	< **0.001**		
Region	16.42	1, 1646.09	< **0.001**		
IHI	98.81	1, 2160.19	< **0.001**		
ICV	231.99	1, 544.55	< **0.001**		
Age	0.07	1, 543.54	0.79		
Sex	1.94	1, 543.71	0.16		
Group:Region	4.84	2, 1646.09	**0.01**		
Group:Hemisphere: Region	1.71	2, 1646.09	0.18		

*AIC* Akaike information criterion, *ICV* intracranial volume, *IHI* incomplete hippocampal inversion.

Bolded values indicate significance at *p* < 0.05.
